# Current approaches for Usher syndrome disease models and developing therapies

**DOI:** 10.3389/fcell.2025.1547523

**Published:** 2025-06-20

**Authors:** Fiona K. Leith, Joey Lye, Derek S. Delaney, Samuel McLenachan, Fred K. Chen, Marcus D. Atlas, Elaine Y. M. Wong

**Affiliations:** ^1^ Hearing Therapeutics, Ear Science Institute Australia, Nedlands, WA, Australia; ^2^ Centre for Ear Sciences, Medical School, The University of Western Australia, Nedlands, WA, Australia; ^3^ Curtin Health Innovation Research Institute, Curtin University, Bentley, WA, Australia; ^4^ Ocular Tissue Engineering Laboratory, Lions Eye Institute Australia, Nedlands, WA, Australia; ^5^ Centre for Ophthalmology and Visual Sciences, The University of Western Australia, Nedlands, WA, Australia; ^6^ Department of Ophthalmology, Royal Perth Hospital, Perth, WA, Australia; ^7^ Ophthalmology, Department of Surgery, University of Melbourne, East Melbourne, VIC, Australia; ^8^ Centre for Eye Research Australia, Royal Victorian Eye and Ear Hospital, East Melbourne, VIC, Australia; ^9^ Curtin Medical School, Faculty of Health Sciences, Curtin University, Bentley, WA, Australia

**Keywords:** Usher syndrome, hearing loss, inner ear, hair cell, gene therapy

## Abstract

Usher syndrome is a severely debilitating autosomal recessive disorder characterised by congenital or progressive hearing loss, gradual vision loss and in some subtypes, vestibular dysfunction. Much progress has been made in recent years in creating appropriate preclinical models for most subtypes of Usher syndrome to facilitate the development of novel therapies. In this review, we provide an update on new preclinical models of Usher syndrome, with a particular focus on induced pluripotent stem cells and new organoid models. An update on the status of novel therapies is provided, including the development of new genetic therapies using new preclinical models and those currently in clinical trials.

## 1 Introduction

**FIGURE 1:**
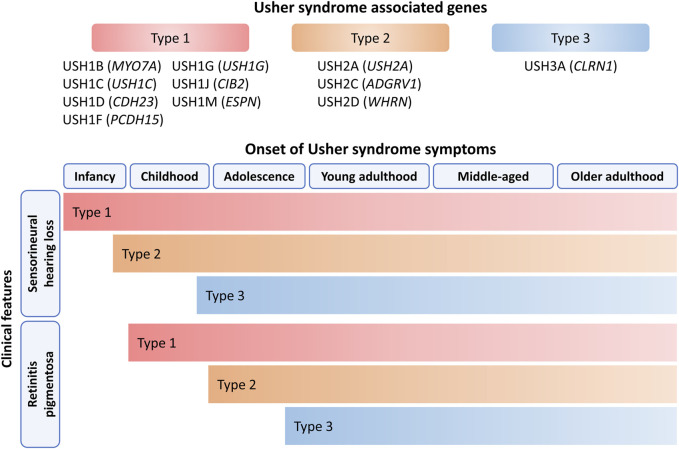
Onset of SNHL and RP symptoms in USH. The age, progression and severity of symptoms depends on the USH type which is determined by the underlying genetic cause. HL is the first clinical feature of USH and progressively deteriorates except for USH1 patients as they are born with severe-to-profound HL. USH2 patients are born with moderate-to-severe HL while USH3 patients are born with normal hearing and HL gradually becomes more severe over time. All USH patients are born with normal eyesight and retinal symptom onset is variable for each USH type. Vision loss typically begins during early childhood to adolescence for USH1 and USH2 patients and progressively worsens over several decades. The mean onset of retinal disease occurs during mid-adolescence for USH3 patients. The age groups were categorised as follows: infant (0–2 years old), childhood (2–11 years old), adolescence (12–18 years old), young adulthood (18–39 years old), middle-aged (40–59 years old) and older adulthood (60 years old and above).

Usher syndrome (USH) is a debilitating autosomal recessive disorder, representing almost 50% of genetic deaf-blindness and has a global prevalence of 4–17 per 100,000 people (Kimberling et al., 2010). The effect on patient morbidity is significant with some subtypes causing severe-to-profound bilateral sensorineural hearing loss (SNHL), profound vision loss due to retinitis pigmentosa (RP) and vestibular dysfunction. The healthcare burden of USH-related RP was predicted to cost up to USD $371–880 million annually in the United States and USD $45–185 million in Canada (Gong et al., 2021). USH is both clinically and genetically heterogenous and has been classified into three USH types based on age of onset, symptom progression and the causative gene. Eleven genes have been associated with USH: *MYO7A, USH1C, CDH23, PCDH15, USH1G, CIB2, ESPN, USH2A, ADGRV1, WHRN* and *CLRN1*, all of which are inherited in an autosomal recessive manner as shown in Figure 1 (Ahmed et al., 2018; Khateb et al., 2018; Whatley et al., 2020).

## 2 Current management and treatment of Usher syndrome

There is no effective cure for any subtype of USH, merely ways to manage or mitigate symptoms. Hearing devices and cochlear implants are the standard of care for USH patients with residual hearing. Unfortunately, in cases of profound HL, cochlear implantation may not be sufficient in improving quality of life. Patients with USH who received late cochlear implantation have better hearing thresholds but improvement in speech recognition remains difficult to achieve (Davies et al., 2021). Most cases of USH are identified through hearing screening tests in newborns, due to early onset of hearing impairment for USH type 1 and 2 cases. Early intervention allows planning of long-term treatment and implementation of supportive strategies including learning speech signals and sign language at prelingual age and physiotherapy for balance issues for USH type 1 (USH1) patients.

There is no approved treatment to reverse the poor vision in dim light and slow down the progressive constricting visual field associated with USH-related RP (French et al., 2020; Zaw et al., 2022). Patients may present with central visual loss associated with cystoid macular oedema. These may respond to carbonic anhydrase inhibitor topically or systemically. However, intraocular injection of steroid implant (Ozurdex) and anti-vascular endothelial growth factor agents have been tried in those with oedema resistant to carbonic anhydrase inhibitors (Chen et al., 2022). Posterior subcapsular cataract is commonly seen in USH and standard cataract surgery in carefully selected patients may restore vision. However, post-operative complications such as cystoid macular oedema, posterior capsular opacification and zonular deficiency are more frequent in patients with RP (Nguyen et al., 2023b; Georgiou et al., 2024).

## 3 Usher syndrome type 1 (USH1)

USH1 is the most severe type and is characterised by severe-to-profound SNHL across all frequencies and vestibular dysfunction at birth followed by prepubertal onset of progressive RP. To date, various mutations in *MYO7A, USH1C, CDH23, PCDH15, USH1G, CIB2* and *ESPN* have been shown to be causative of USH1 (Whatley et al., 2020; Fuster-García et al., 2021; Nisenbaum et al., 2022).

### 3.1 Usher syndrome type 1B (MYO7A)

*MYO7A* is located on chromosome 11q13.5 and has been linked to cause both USH1B and non-syndromic HL. The prevalence of *MYO7A-*associated HL has been reported across various populations with the lowest at 1.79% and the highest at 5.7% (Sloan-Heggen et al., 2016; Baux et al., 2017; Abu Rayyan et al., 2020). Recently, Ma et al. (2023) found that of 879 patients in Yunnan, China with HL onset before 6 years of age, 4.9% had a mutation in *MYO7A*. Another study by Watanabe et al. (2024) have identified 1.36% of HL patients within a Japanese cohort having *MYO7A*-associated HL, moreover, the prevalence of USH1B within autosomal recessive or sporadic HL patients was 0.32%.

**FIGURE 2:**
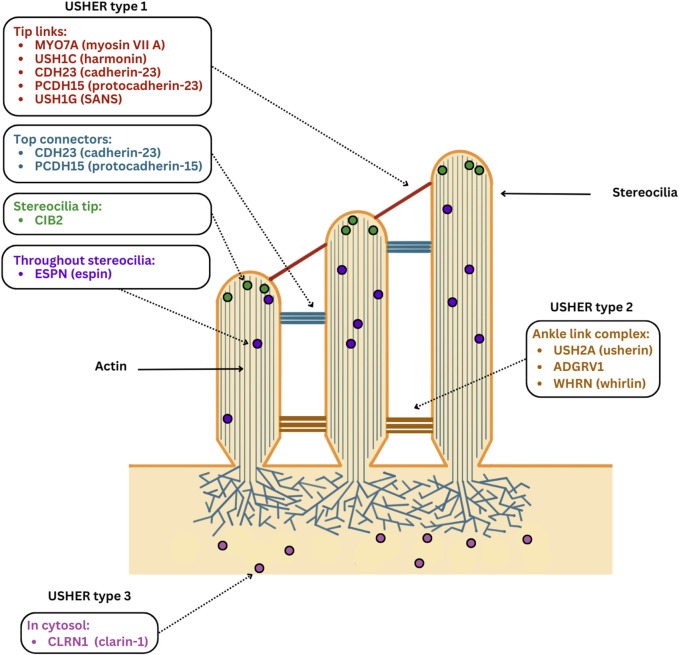
Localisation of USH proteins in HCs. USH1 proteins are mainly localised in upper regions of HC. Myosin VIIA and harmonin contribute to the upper tip link density in HC, while protocadherin-23 and cadherin-23 form the lower and upper sections of tip links with associations to top connectors during development. SANS assembles with other USH1 proteins at stereocilia tips forming complexes, whereas CIB2 is concentrated on the apical surfaces of HC in addition to the tips of stereocilia. Meanwhile espin plays a role in stereocilia lengthening during development. USH2 proteins are primarily confined to the ankle link complex, with usherin being demonstrated to form the complex, ADGRV1 facilitating ankle link formation and whirlin playing a role in establishing the ankle link complex. Clarin-1, implicated in USH3, is localised to the cytosol and is concentrated on the apical and basal ends of HCs.

The MYO7A protein is a member of the myosin family, essential for the development and maintenance of hair cell (HC) stereocilia in the cochlea. It is localised to the upper tip link density of HC stereocilia (Figure 2) where it complexes with harmonin, SANS and CDH23 to facilitate mechanotransduction and maintain tension of the tip link (Grati and Kachar, 2011; Li et al., 2020). During development it is thought that a complex of MYO7A and PCDH15 at the base of stereocilia play a role in setting stereocilia polarity and cohesion, while MYO7A plays a determining factor in stereocilia height (Senften et al., 2006; Prosser et al., 2008). *MYO7A* mutations result in HC with disorganised stereocilia in animals, with abnormalities in size and orientation. Several studies have also reported that *Myo7a Ewaso* mice, p.Ile487Asn, exhibit HC loss in the cochlea (Miller et al., 2012; Calabro et al., 2019).

In the retina, MYO7A is mainly found in the apical region of retinal pigment epithelia (RPE) where it plays a key role in protein localisation of opsin and melanosomes, and transportation of phagosomes from the apical to basal RPE (Williams and Lopes, 2011). Within photoreceptors, MYO7A is localised to the ciliary and periciliary membranes (Liu et al., 1999; Gibbs et al., 2004). In an USH1B retinal organoid model, Leong et al. (2022) detected *MYO7A* expression in Muller and bipolar cells by single-cell RNA sequencing but were unable to detect protein localisation.

Tissue-specific functions of MYO7A are mediated by the expression of certain isoforms. There are currently two reported isoforms in the inner ear and two isoforms in the retina (Li et al., 2020; Gilmore et al., 2023). The canonical isoform was predominantly found in all inner hair cells (IHCs), whereas in outer hair cells (OHCs) its expression is highest in the apex and decreases towards the base of the cochlea (Li et al., 2020). The second isoform differs from the canonical isoform by the lack of 11 amino acids in the N-terminal extension of the motor head domain. While this shortened isoform is primarily expressed in OHCs and weakly expressed in IHCs, the expression in OHCs is thought to be in a tonotopic gradient inverse to that of the canonical isoform (Li et al., 2020). A recent study confirmed that the canonical isoform acts as a tip link motor to control tensioning of the mechanotransduction channel (Underhill et al., 2025).

### 3.2 Usher syndrome type 1C (USH1C)

Mutations in *USH1C* account for 2% of USH cases. *USH1C* is located on chromosome 11p15.1 and encodes the structural protein, harmonin. *USH1C* consists of 28 exons, with 20 that are conserved and 8 that are alternatively spliced to encode multiple isoforms categorised into three splice groups; a, b and c (Verpy et al., 2000). Mutations in domains shared across isoforms, N-harm, PDZ, or Coiled-coil (CC) domains, are more associated with USH1C (Grotz et al., 2022).

Harmonin is a PDZ-containing scaffold protein that is involved in the development, maintenance and excitation of sensory cells in the inner ear and retina by interacting with other USH genes. In the inner ear, harmonin isoform b is localised to the upper tip link density of the HC stereocilia where it interacts with SANS and MYO7A (Yan et al., 2022). Harmonin binds to CDH23 at the PDZ2 domain, the disruption of which deleteriously affects hair bundle development. Additionally, the CC and Proline-, Serine- and Threonine-rich (PST) domains with a known F-actin bundling function, when disrupted, did not affect development of the stereocilia bundle but did affect the transducer adaptability and displacement sensitivity (Grillet et al., 2009; Yan et al., 2022). Harmonin is additionally localised to the ribbon synapse of IHC and has been suggested to modulate Cav1.3 channels via ubiquitination (Gregory et al., 2011).

Several harmonin isoforms are present in the retina. Subclasses a and c are present in the inner and outer segments of photoreceptors as well as the synaptic terminal, while expression of subclass b has been reported in the outer segment (Reiners et al., 2003). Harmonin colocalises with CDH23 in the photoreceptor inner segment, and with CDH23, PCDH15 and MYO7A at the ribbon synapse where these proteins may contribute to cell adhesion and endo- or exocytosis. Previous studies have shown that the co-localisation and interaction of the USH1 proteins occurs at the ribbon synapses in the retina and cochlear HCs, however the role of USH1 proteins in the synaptic structure of retina and cochlea are still unclear (Reiners et al., 2005; Phillips et al., 2011; Miles et al., 2021). Harmonin is known to have a role in shaping cochlear stereocilia through *β*-catenin signalling during development of the inner ear (Boëda et al., 2002; Schäfer et al., 2023).

### 3.3 Usher syndrome type 1D (CDH23)

The *CDH23* gene contains 69 exons that spans across 300 kb and it encodes for the cadherin 23 protein (CDH23) (Bork et al., 2001). Mutations in *CDH23* results in USH1D and account for approximately 20% of USH1 cases making it the second most common cause of USH1 (Oshima et al., 2008). Cadherins are a family of calcium-dependent cell adhesion transmembrane proteins. Three subclasses of CDH23 isoforms are predominantly expressed in the inner ear and retina: A, B and C. Isoform A is the full-length protein consisting of an extracellular domain containing 27 extracellular cadherin (EC) repeats, a transmembrane domain and the cytoplasmic domain containing two PDZ-binding interfaces (Lagziel et al., 2005; Vanniya et al., 2018). Isoform A forms the stereocilia tip links with PCDH15 (Kazmierczak et al., 2007). Moreover, they are components of kinociliary links and are associated with transient lateral links that connects neighbouring stereocilia during development (Richardson and Petit, 2019). The specific role of isoforms B and C in the inner ear and retina is uncertain. Mutations in *CDH23* cause disorganised HC stereocilia with misplaced kinocilia, resulting in HL and vestibular dysfunction (Palma et al., 2001). In the retina, CDH23 is found in the outer segment and calyceal processes of photoreceptors where, along with PCDH15, it is thought to have a role in the structural organisation of the outer segment (Schietroma et al., 2017).

### 3.4 Usher syndrome type 1F (PCDH15)

Protocadherin 15 (*PCDH15*) is the gene implicated in USH1F, which accounts for 11%–19% of USH1, however *PCDH15* mutations are responsible for 50%–60% of USH1 cases within the Ashkenazi Jewish population (Yusuf et al., 2022). *PCDH15* is 980 kb long and contains 33 exons, with mutations in this gene also being responsible for non-syndromic HL DFNB23 (Ahmed et al., 2001; Nisenbaum et al., 2022). In the inner ear, CDH23 and PCDH15 form the upper and lower sections of the tip link respectively and are components of transient links and kinocilial links (Kazmierczak et al., 2007; Richardson and Petit, 2019). At the lower tip link density of the stereocilia, PCDH15 binds to the mechanotransduction channel pore-forming subunits TMC1 and TMC2 (Maeda et al., 2014).

The PCDH15 protein has three isoforms: A B and C, of which isoform A is the longest, consisting of extracellular domains containing 11 EC repeats, a transmembrane domain and cytoplasmic domain. Isoform A has three subtypes (CD1, CD2 and CD3) which vary in cytoplasmic domain length (Pepermans et al., 2014). Ahmed et al. (2003) described isoform B containing a shortened extracellular domain with less EC repeats, a transmembrane domain and cytoplasmic domain. Isoform C lacks the transmembrane domain and cytoplasmic domain and is thought to be secreted, possibly acting as a ligand for a membrane receptor (Rouget-Quermalet et al., 2006). Studies by Pepermans et al. (2014) and Webb et al. (2011) reported that while *Pcdh15* knockout mouse models for isoform CD1 and CD3 do not have HL, mice with isoform CD2 knocked out presented with functional and morphological HC defects. This indicates that the CD2 isoform is essential for hearing in humans which was shown in a study where the *PCDH15* p.P1515Tfs*4 mutation only affected isoform CD2 in profoundly deaf children from two unrelated families (Pepermans et al., 2014). In the retina, PCDH15 is found in the outer segment and calyceal processes of photoreceptors (Schietroma et al., 2017).

### 3.5 Usher syndrome type 1G (SANS)

Mutations in *USH1G* account for up to 7% of USH1 cases (Nisenbaum et al., 2022). *USH1G* is located on chromosome 17q25.1 and encodes a scaffold protein containing ankyrin repeats and a sterile alpha motif (SAM) domain (SANS) protein which consists of three ankyrin repeats, a central domain followed by a sterile alpha motif domain and a class I PDZ-binding motif. Overlack et al. (2008) showed SANS was expressed in the photoreceptor cell layer and the inner and outer plexiform layer. SANS is localised within the ciliary apparatus and synapse of photoreceptor cells where it binds with usherin and whirlin and interconnects the complex with the microtubule cytoskeleton to facilitate transportation processes (Overlack et al., 2008; Sorusch et al., 2017). Recently, Yildirim et al. (2021) and Fritze et al. (2023) showed that SANS interacts with spliceosomal proteins such as SF3B1, PRPF6 and PRPF31 in Cajal bodies and nuclear speckles. This suggests SANS regulates pre-mRNA splicing of USH genes and other genes related to cellular proliferation.

### 3.6 Usher syndrome type 1J (CIB2)

*CIB2* encodes for calcium and integrin binding protein 2 (CIB2), a DNA-dependent protein kinase interacting protein also associated with the non-syndromic HL DFNB48. Three isoforms of CIB2 have been identified in humans (Riazuddin et al., 2012). In the inner ear, CIB2 is localised to the tip of stereocilia and apical surfaces of HCs (Michel et al., 2017). Moreover, Riazuddin et al. (2012) demonstrated that CIB2 interacts with MYO7A and whirlin, and hypothesised several functions: to regulate calcium during mechanotransduction, photoreceptor maintenance and homeostasis. In *Cib2* knockout mice, mechanotransduction in cochlear HC but not vestibular HC was reduced due to CIB3 acting as a redundancy for CIB2 function (Michel et al., 2017; Wang et al., 2017; Liang et al., 2021). In the retina, *CIB2* is expressed by RPE, photoreceptors and certain ganglion cells. Sethna et al. (2021) demonstrated that loss of CIB2 in the mice RPE resulted in a phenotype similar to that of age-related macular degeneration and dysregulation of phagolysosomal processing.

Some studies have expressed scepticism of *CIB2* as being causative of USH1. Booth et al. (2018) found no patients with RP in their investigation of six families across three ethnicities with a mutation in *CIB2* during ophthalmological evaluation. Similarly, murine models differ in phenotype, a lack of behaviour characterising vestibular dysfunction and changes in stereocilia morphology inconsistent with other USH mouse models. Moreover, a meta-analysis of 11 next-generation sequencing studies of patients with USH and 21 next-generation sequencing studies of patients with isolated deafness found no mutations of CIB2 in subjects with HL and visual impairment (Jouret et al., 2019).

### 3.7 Usher syndrome type 1M (ESPN)

*ESPN* was recently associated with USH1M in a study where a consanguineous Pakistani family with prelingual SNHL, vestibular dysfunction and progressive vision impairment were identified to have an in-frame deletion within the gene (Ahmed et al., 2018). *ESPN* is also associated with the human deafness locus *DFNB36* (Naz et al., 2004). *ESPN* encodes for the actin-bundling, cytoskeletal regulatory protein espin. Espin has a critical role in the inner ear for stereocilia lengthening during development (Donaudy et al., 2006). In the retina, espin is expressed in the outer limiting membrane, localised to the inner segment and the calyceal processes (Wang et al., 2011a). It is also thought to be present in the microvilli of Muller cells (Sekerková et al., 2004). Espin and the USH2 protein whirlin have been found to colocalise and interact at the ankle link of cochlear stereocilia and at the periciliary membrane complex of photoreceptors (Wang et al., 2011a).

## 4 Usher syndrome type 2 (USH2)

USH type 2 (USH2) is the most common type of USH and accounts for more than 60% of USH cases (Castiglione and Möller, 2022). USH2 patients are born with moderate-to-severe SNHL with progressive vision loss in the second decade of life and very rarely present with balance issues (Magliulo et al., 2017; Wafa et al., 2021). USH2 is characterised by congenital SNHL with a down-sloping audiometric configuration in which HL at low frequencies is typically mild-to-moderate and gradually becomes more severe at higher frequencies (Abadie et al., 2012). USH2 patients have variable onset of RP, which typically develops during adolescence and progressively worsens, though patients aged 5 years with RP have also been described (Sadeghi et al., 2006).

### 4.1 Usher syndrome type 2A (USH2A)

Mutations in the *USH2A* gene were predicted to be involved in 55%–90% of USH2 cases (Millán et al., 2011; Jouret et al., 2019). *USH2A* is located on chromosome 1q41 and spans over ∼800 kb. Usherin is a 5,202 amino acid transmembrane protein encoded by 72 exons (Eudy et al., 1998). There are two usherin isoforms: the short extracellular and long transmembrane isoform. The short isoform is encoded by 21 exons and contains a signal peptide, a laminin-globular-like domain, a laminin N-terminus domain, 10 laminin-epidermal growth factor domains and four fibronectin type III (FN3) domains. The long isoform is encoded by another 51 exons with two additional laminin globular domains and 28 FN3 domains in its N-terminus, followed by a transmembrane domain and an intracellular PDZ-binding motif (PBM) domain at the C-terminal tail.

In the developing cochlea, usherin is expressed in the ankle link (Figure 2) and spiral ganglion cells. Usherin forms the ankle link complex, a transient structure that connects and supports the growing stereocilia in immature HCs (Liu et al., 2007; Zou et al., 2015; Wang et al., 2023). In the retina, usherin is expressed in the connecting cilium and was recently proposed to facilitate ciliary trafficking of intracellular protein components in the inner segment of photoreceptors (Toms et al., 2020; Crane et al., 2023; Tebbe et al., 2023).

### 4.2 Usher syndrome type 2C (ADGRV1)

*ADGRV1* is the fourth most commonly mutated gene in USH and is located on chromosome 5q14.3-21.3 (Jouret et al., 2019). *ADGRV1* contains 90 exons, spanning over 600 kb of genomic DNA and encodes for Adhesion G protein-coupled Receptor V1 (ADGRV1; also known as Very Large G protein-coupled Receptor-1). There are three major alternatively spliced transcripts identified in humans, each encoding different isoforms of various lengths termed ADGRV1a, ADGRV1b and ADGRV1c. ADGRV1b is the largest full-length isoform, containing all 6,306 amino acids and is the predominant form in the inner ear and retina. At the N-terminal, its extracellular portion consists of 35 sodium-calcium exchangers (Calxβ) domains, one laminin-G/pentraxin domain and seven epilepsy-associated repeat (EAR) domains located in between the 22-23rd Calxβ domain. At the intracellular carboxyl end, there is a G-protein-coupled proteolytic site (GPS) domain, seven-transmembrane (7TM) domain and a PBM domain. ADGRV1a (1967 amino acids) and ADGRV1c (2296 amino acids) are shorter isoforms without the signal transduction 7TM domain due to partial deletion of exon 31.

Like other USH2 genes, the ADGRV1 protein is essential for ankle link formation during cochlear HC development (McGee et al., 2006; Liu et al., 2007). In the developing retina, *ADGRV1* is highly expressed in neural retinal precursors and in mature RPE (McMillan et al., 2002). A recent study revealed ADGRV1 and CIB2 co-localised in the synaptic and ciliary region of photoreceptor cells. Linnert et al. (2023) demonstrated their interaction with ciliary proteins, such as the TRiC/CCT chaperonin complex, to facilitate cargo transportation from the inner to the outer segment of photoreceptors. Along with other recent studies, ADGRV1 is involved in focal adhesions for mechanosensing during cell migration, as demonstrated by the absence of ADGRV1 resulting in autophagy (Kusuluri et al., 2021; Linnert et al., 2023).

### 4.3 Usher syndrome type 2D (WHRN)

USH2D is caused by pathogenic *WHRN* variants and is the least prevalent USH2 subtype. The *WHRN* gene is located on chromosome 9q32-34 and contains 12 exons that encode whirlin, a 907 amino acid protein. The full-length whirlin isoform is highly expressed in cochlear HCs and retinal photoreceptors, while the shorter isoform is only present in HCs and is controlled by a different promoter (Mburu et al., 2003). The long whirlin isoform consists of an Ala/Gly/Ser-rich region at the N-terminal end, followed by a harmonin-homology domain (HHD1), two PDZ domains (PDZ1, PDZ2), a second HHD (HHD2), a proline-rich region, a third PDZ domain (PDZ3) and a PBM domain at its C-terminal end. The PDZ1 domain of the long isoform has been shown to interact with the C-terminal tails of cadherin 23 and protocadherin 15 (Michel et al., 2020). The short isoform consists of HHD1, PDZ1, PDZ2 and HHD2, and was proposed to be involved in polymerisation and stabilisation of actin filaments at the tips of the tallest stereocilia with stereociliary components Eps8 and Myosin XVa for stereocilia elongation (Manor et al., 2011).

Similar to other USH2 proteins, the PDZ1 and PDZ2 domain of whirlin interacts with the PBM domain of usherin and ADGRV1 to establish the ankle link complex, where whirlin acts as a scaffold to connect the neighbouring stereocilia with other USH2 proteins (van Wijk et al., 2006; Chen et al., 2014; Guan et al., 2023). Moreover, whirlin has been shown to interact with Esp8 and myosin XVa in cochlear HCs, in which they are essential for regulating stereocilia growth during development (Mburu et al., 2006; Wang et al., 2011a). In the retina, whirlin recruits other USH2 proteins to the periciliary membrane complex in the photoreceptors (Zou et al., 2011).

## 5 Usher syndrome type 3 (USH3)

USH type 3 (USH3) is the rarest form of USH and exhibits the most phenotypic heterogeneity. USH3 accounts for 1%–6% of worldwide USH cases, however it is significantly more prevalent among Finnish and Ashkenazi Jewish populations. In these populations, USH3 accounts for up to 40% of total USH cases (Ness et al., 2003; Plantinga et al., 2005; Herrera et al., 2008). As for its clinical presentation, USH3 typically features a later onset of the classic USH symptoms compared with USH1 and USH2. HL in USH3 patients is progressive, being mostly diagnosed by the age of 10, though onset has been observed to occur as late as 35 (Ness et al., 2003; Plantinga et al., 2005). As for visual function, RP generally occurs from late adolescence to the fourth decade of life and is also progressive, with patients experiencing total or near-total blindness past the age of 50 (Ness et al., 2003; Herrera et al., 2008; Yoshimura et al., 2015). Vestibular dysfunction is variable, occurring in approximately half of USH3 patients (Sadeghi et al., 2005; Wafa et al., 2021).

*CLRN1* is the gene implicated in USH3 and encodes clarin-1, a membrane protein generally involved in organisation of cilia and F-actin in the cytoskeleton (Adato et al., 2002; Herrera et al., 2008; Tian et al., 2009; Ratnam et al., 2013). *Clrn1* expression has been demonstrated to occur in HCs in the apical region (Figure 2) and at the base near the ribbon synapse in mice, zebrafish and non-human primate models (Zallocchi et al., 2009; Ogun and Zallocchi, 2014). In the retina it is expressed by Muller cells, making it unique among the USH proteins as it is not expressed by photoreceptors (Xu et al., 2020). Mutations in *CLRN1* generally affect the clarin-1 protein by impeding its trafficking to the plasma membrane (Isosomppi et al., 2009; Ogun and Zallocchi, 2014; Gopal et al., 2015; Geng et al., 2017). The most common mutation in North American and Ashkenazi Jewish populations is the c.143T>C mutation causing a N48K substitution in the clarin-1 protein (Herrera et al., 2008; Ratnam et al., 2013). This has been demonstrated to affect glycosylation of the final protein, resulting in reduced trafficking to HC apices and reduced stability (Gopal et al., 2015).

## 6 USH-related gene modifier (PDZD7)

PDZ-domain containing 7 (PDZD7) is a large structural protein and paralog of WHRN. The *PDZD7* gene spans 23.3 kb on chromosome 10 and comprises 16 exons, while the protein consists of three PDZ-like domains and a HHD which is found between the second and third PDZ domains. PDZD7 forms part of the transient ankle link complex at the base of hair bundles with usherin, ADGRV1 and whirlin (Grati et al., 2012; Chen et al., 2014; Du et al., 2020; Guan et al., 2023). It has also been observed to interact with myosin VIIa and other proteins forming the stereocilia (Morgan et al., 2016). PDZD7 has two isoforms: a short isoform exclusively expressed in the cytoplasm of HCs; and a long isoform, which is also localised to cochlear hair bundles and is part of ankle link complexes (Du et al., 2020). PDZD7 protein expression in the retina peaks prenatally and is almost undetectable in the mature retina (Zou et al., 2013; Yang et al., 2014). Zou et al. (2013) generated mice with digenic heterozygous mutations in *PDZD7* with *USH2A*, *ADGRV1*, *WHRN* or *SANS* and did not observe any effect on hearing function. This was despite demonstrating mice harbouring homozygous *PDZD7* mutations disrupting the localisation of USH2 protein complex to the hair bundle (Zou et al., 2013). These mice exhibited profound HL and malformed hair bundles without vestibular dysfunction or RP.

Other studies examining the physical interaction between PDZD7 and USH proteins have suggested that mutations in *PDZD7* could modify the phenotype of USH2 patients due to the role of PDZD7 in bridging the ankle link proteins (Grati et al., 2012; Chen et al., 2014; Morgan et al., 2016; Lin et al., 2021). However, a few studies have reported non-syndromic HL caused solely by mutations in PDZD7, which have undermined its association with USH in recent years (Booth et al., 2015; Guan et al., 2018; Fahimi et al., 2021).

## 7 Digenic inheritance

Possible digenic inheritance has been reported in some families with mutations in USH genes. Three USH1 patients from a study by Zheng et al. (2004) harboured pathogenic monoallelic mutations for *CDH23* and *PCDH15*; these patients exhibit congenital profound deafness and vestibular dysfunction, accompanied with progressive vision loss. Through genetic screening, family members of the affected USH1 patients were used to confirm the variant origin and each parent was heterozygous for either *CDH23* or *PCDH15*. Yoshimura et al. (2014) also reported digenic inheritance caused by USH1 mutations in *MYO7A* and *PCDH15* in the Japanese population. Other combinations of pathogenic mutations were identified for biallelic mutations in *MYO7A* and monoallelic mutation in *CDH23*, attributing to more severe USH phenotype where earlier onset of RP symptoms such as night blindness was described (Yoshimura et al., 2014). Another study showed digenic inheritance of *PCDH15* and *USH1G* which have been identified in five family members from a Pakistani consanguineous family (Schrauwen et al., 2018). These individuals were presented with non-syndromic HL and no visual or vestibular abnormalities, similar to double heterozygous *Pcdh15* ^*+/av−3J*^ and *Ush1g* ^*+/js*^ mice thus suggesting true digenism for *PCDH15-USH1G* (Zheng et al., 2012; Schrauwen et al., 2018).

Digenic inheritance has also been described for USH2, where a single pathogenic variant was identified in either *ADGRV1* or *PDZD7* presented with mild disease phenotype (Ebermann et al., 2010). Another patient with a homozygous truncation mutation in *USH2A* and a heterozygous frameshift mutation in *PDZD7* was observed to have earlier onset and more severe RP compared to her sister who carried the homozygous *USH2A* mutation but not the *PDZD7* mutation (Ebermann et al., 2010). However, this digenic form of USH2 has not been recapitulated in mice models with heterozygous mutations in *PDZD7* and either USH2 genes or *SANS* (Zou et al., 2013).

## 8 Emerging USH genes

*HARS* has previously been implicated in USH3 and encodes a class IIa aminoacyl tRNA synthetase that loads tRNA with histidine (Abbott et al., 2017). Although *HARS* is ubiquitously expressed throughout the body, for unknown reasons pathological HARS variants are exclusively associated with rare neuropathies. A study by Puffenberger et al. (2012) was the first to identify the *HARS* c.1361A>C (p.Y454S) homozygous variant and associate it to USH3. The *HARS* variant was identified in an Amish Plain population, in which the patients exhibited severe retinal dystrophy and cone dysfunction from birth and later onset HL in childhood. The disorder was present in 1.5% of this population. This variant is also linked to damaged afferent sensory cells, predisposing affected individuals to episodic psychosis and sudden death through unknown mechanisms (Puffenberger et al., 2012). While the variable deaf-blindness severity and onset observed with this *HARS* variant could warrant its association with USH3, the additional symptoms observed in affected individuals complicate the designation of *HARS* as a *bona fide* USH gene. The number of reports on this *HARS* variant are also limited.

Recently several clinical reports of patients with *ARSG* mutations in have been proposed as Usher syndrome type 4 (USH4). These patients were presented with a combination of late-onset RP and SNHL (Khateb et al., 2018; Abad-Morales et al., 2020; Fowler et al., 2021; Peter et al., 2021; Velde et al., 2022; Bauwens et al., 2025). In the animal study, there is a clear phenotypic change in *Arsg* ^*−/−*^ mice which they were characterised with progressive retinal degeneration at 1–6 months of age (Kruszewski et al., 2016). There are limited reports on *ARSG* in the inner ear, though Girotto et al. (2014) revealed restricted ARSG expression at the apical side of inner and outer HCs of P5 mice cochlea. Despite the hints of *ARSG* reported in current literature, its precise functional role in the inner ear and retinal sensory cells remains too early to be a causative USH gene.

## 9 Preclinical animal models

There are various animal models that exist for the study of USH. Up until recently, animal models were limited to mice and some zebrafish models (Williams, 2008). The current available animal models for USH are too numerous to discuss individually and have been listed in Table 1. The earlier USH mouse models were characterised by their tendency to run in circles and head tossing, which arise from vestibular dysfunction caused by their mutation. They are even named as such, some examples include *shaker-1* (USH1B), *deaf circler* (USH1C), *waltzer* (USH1D), *Ames waltzer* (USH1F), *Jackson shaker* (USH1G) and *whirler* (USH2D). Aside from the latter example, most mouse models for USH2 do not present with vestibular dysfunction, reflecting the human disease.

**TABLE 1:** Preclinical animal models used for the study of USH disease mechanisms and therapies. Only the homozygotic phenotypes are reported here.

Causative gene	Model	Auditory phenotype	Vision phenotype	Vestibular phenotype	Reference
MYO7A	*Dumbo* mouse	Severe progressive HL, progressive hair bundle disorganisation (apical to basal)	Normal	Normal	Miller et al. (2012)
MYO7A	*Ewaso* mouse	Profound HL, collapsed organ of Corti, progressive hair bundle disorganisation (basal to apical)	Normal	Hyperactivity, circling, irregular hair bundle organisation	Miller et al. (2012)
MYO7A	*Headbanger* mouse	Disorganised stereocilia bundles (more prominent at apex), raised compound action potential thresholds	Not reported	Hyperactivity, head bobbing, wispy stereocilia	Rhodes et al. (2004)
MYO7A	*Polka* mouse	No auditory startle response, elevated ABR thresholds, no DPOAE response, disorganised hair bundles	No retinal degeneration, mislocalisation of melanosomes	Circling, poor performance in swim tests	Schwander et al. (2009)
MYO7A	*Shaker-1* mouse	Progressive degeneration of organ of Corti	No retinal degeneration, mislocalisation of melanosomes	Head tossing and circling	Gibson et al. (1995); Liu et al. (1998)
MYO7A	*Myo7a* ^*4494SB*^ mouse	Abnormal HC morphology and physiology	No retinal degeneration, mislocalisation of opsins, disrupted phagocytosis in RPE	Head tossing and circling	Mburu et al. (1997); Liu et al. (1999)
MYO7A	*Myo7a* ^*6J*^ mouse	Profound HL, progressive disorganisation of hair bundles, no gross electrophysiological response	No retinal degeneration	Head tossing and circling	Gibson et al. (1995); Kros et al. (2002); Self et al. (1998)
MYO7A	*Myo7a* ^*816SB*^ mouse	Profound HL, progressive disorganisation of hair bundles, no gross electrophysiological response	No retinal degeneration, reduced electrophysiological response	Head tossing and circling	Gibson et al. (1995); Self et al. (1998)
MYO7A	*Myo7a* ^*26SB*^ mouse	Progressive disorganisation of hair bundles, no electrophysiological response	No retinal degeneration, reduced electrophysiological response	Head tossing and circling	Mburu et al. (1997)
MYO7A	*Myo7a* ^*3336SB*^ mouse	Progressive disorganisation of hair bundles, no electrophysiological response	Retinal degeneration unrelated to *Myo7a* gene	Head tossing and circling	Mburu et al. (1997); Libby and Steel (2001)
MYO7A	*Myo7a* ^*4626SB*^ mouse	Progressive hair bundle disorganisation, reduced mechanochanotransduction activity	No retinal degeneration, mislocalisation of opsins, reduced electrophysiological response	Not reported	Rinchik and Carpenter (1999); Kros et al. (2002)
MYO7A	*MYO7A* knockdown by CRISPR in rhesus macaque primate	Minimal ABR threshold or DPOAE response at 1 month, increasing sensitivity with age	Normal vision; slightly thinner inner/outer segment layer	Not reported	Ryu et al. (2022)
MYO7A	*mariner* ^*ty220D*^ zebrafish	Lack of acoustic vibrational sensitivity, splaying of stereocilia	Not reported	Not reported	Ernest et al. (2000)
MYO7A	*mariner* ^*tc320b*^ zebrafish	Lack of acoustic vibrational sensitivity, splaying of stereocilia	Not reported	Not reported	Ernest et al. (2000)
MYO7A	*mariner* ^*tn4503*^ zebrafish	Lack of acoustic vibrational sensitivity, splaying of stereocilia	Not reported	Not reported	Ernest et al. (2000)
MYO7A	*mariner* ^*tn3540*^ zebrafish	Lack of acoustic vibrational sensitivity, less severe splaying of stereocilia than aforementioned models	Not reported	Not reported	Ernest et al. (2000)
MYO7A	*mariner* ^*tr202b*^ zebrafish	Lack of acoustic vibrational sensitivity, fewer splayed hair bundles	Not reported	Not reported	Ernest et al. (2000)
USH1C	*Deaf circler* (BALB/c) mouse	Completely deaf, progressive loss of HCs followed by loss of spiral ganglion cells	Normal vision, slight peripheral retinal degeneration	Head tossing, circling	Johnson et al. (2003)
USH1C	*Deaf circler 2 jackson* (C57BL/6J) mouse	Completely deaf, progressive loss of HCs followed by loss of spiral ganglion cells	Normal	Head tossing, circling	Johnson et al. (2003)
USH1C	*Ush1c* ^*−/−*^ mouse	Deviated kinocilium, hair bundle fragmentation and misorientation	Not reported	Not reported	Lefevre et al. (2008)
USH1C	*Ush1c* c.216G>A knock-in mouse	Profound deafness, disorganised stereocilia, loss of middle and basal HCs	Abnormal electroretinogram, progressive rod photoreceptor death	Not reported	Lentz et al. (2010)
USH1C	*ush1c* ^*fh293*^ zebrafish (nonsense mutation in exon 5)	No auditory startle response, disorganised hair bundles, reduced harmonin distribution	Low response to optokinetic assay, depletion of Muller cells, decreased photoreceptor ribbon synapse function	Circling behaviour	Phillips et al. (2011)
USH1C	Zebrafish (morpholino-based knockdown of exon 2)	No auditory startle response, disorganised hair bundles, reduced harmonin distribution	Low response to optokinetic assay, depletion of Muller cells, decreased photoreceptor ribbon synapse function	Circling behaviour, disorganised hair bundles, reduced harmonin distribution	Phillips et al. (2011)
USH1C	*USH1C* c.91C>T (p.R31X) (pig)	Elevated ARB thresholds at 3 weeks	Early onset retinal degeneration, disrupted photosensitive disc architecture in photoreceptor outer segments	Circling behaviour, balance difficulties	Grotz et al. (2022)
CDH23	*Waltzer* mouse	Disorganised hair bundles, minimal ABR response	Not reported	Circling behaviour; thicker, disorganised and fused stereocilia	Palma et al. (2001)
CDH23	*Sputnik* zebrafish	Splayed hair bundles, absent CDH23 localisation, no formation of tip links, reduced mechanotransduction activity	Not reported	Vestibular dysfunction	Nicolson et al. (1998); Söllner et al. (2004)
CDH23	*Xenopus tropicalis* (morpholino-based knockdown)	Not reported	Distorted morphology of outer segment, absent calyceal processes and decreased F-actin	Not reported	Schietroma et al. (2017)
PCDH15	*Ames waltzer* mouse	No behavioural response to sound, no ABR, disorganised stereocilia and neuroepithelia	Not reported	Circling behaviour, disorganised stereocilia	Ahmed et al. (2008)
PCDH15	*Noddy* mouse	Lack of auditory startle reflex, hearing loss	Not reported	Head-bobbing	Geng et al. (2013)
PCDH15	*orbiter* zebrafish	Lack of acoustic and vibrational startle reflex, normal HC morphology	Not reported	Circling behaviour, potentiated light dorsal reflex	Nicolson et al. (1998)
PCDH15	*Xenopus* tropicalis (morpholino-based knockdown)	Not reported	Distorted morphology of outer segment, absent calyceal processes and decreased F-actin	Not reported	Schietroma et al. (2017)
SANS	*Jackson shaker* mouse	Disorganisation of stereocilia bundles, no ABR response	Not reported	Head tossing and circling	Kikkawa et al. (2003)
CIB2	Zebrafish (morpholino-based knockdown)	No response to acoustic stimuli, reduced neuromast cells	Not reported	Could not maintain orientation	Riazuddin et al. (2012)
CIB2	*Drosophila melanogaster* (RNAi-mediated knockdown)	Not applicable	Reduced phototransduction, unable to track fast light stimulus, light dependent retinal degeneration	Not applicable	Riazuddin et al. (2012)
ESPN	*Jerker* mouse	Profound deafness, progressive stereocilia degeneration and HC loss	Not reported	Hyperactivity, circling, head tossing	Zheng et al. (2000)
USH2A	*Ush2a* ^*−/−*^mouse	Moderate-to-severe SNHL at high frequencies from birth, fragmented stereocilia of OHCs	Mild late-onset retinal degeneration	Normal	Liu et al. (2007)
USH2A	*ush2a* ^*rmc1*^ zebrafish	Not reported	Complete absence of usherin, reduced localisation of whirlin and ADGRV1, light-dependent induction of photoreceptor apoptosis, decreased electroretinogram response	Not reported	Dona et al. (2018)
USH2A	*ush2a* ^*b1245*^ zebrafish	Not reported	Usherin present at periciliary membrane, reduced localisation of whirlin and ADGRV1, light-dependent induction of photoreceptor apoptosis, decreased electroretinogram response	Not reported	Dona et al. (2018)
USH2A	*ush2a* ^*hzu6*^ zebrafish	Congenital hearing impairment	Progressive rod and cone photoreceptor degeneration	Not reported	Han et al. (2024)
USH2A	*ush2a* ^*u507*^ zebrafish	Not reported	Increased photoreceptor apoptosis, mislocalised rhodamine	Not reported	Toms et al. (2020)
USH2A	c.2299delG knock-in by CRISPR-Cas9 in mouse	Congenital HC, progressive stereocilia bundle disorganisation in IHCs (apical to basal), elevated ABR thresholds at lower frequencies, mislocalisation of usherin and other USH2 proteins (Adgrv1 and whirlin)	Late-onset progressive retinal degeneration, restricted mutated usherin in inner segments, mislocalisation of opsins, USH2 proteins (Adgrv1 and whirlin)	Not reported	Aller et al. (2010); Crane et al. (2023); Tebbe et al. (2023)
USH2A	*Ush2a exonΔ12* rabbit	Slow progressive moderate-to-severe HL at all frequencies (tested by ABR)	Progressive retinal degeneration, abnormal inner and outer segment and RPE, reduced rod and cone response	Not reported	Nguyen et al. (2023a)
ADGRV1	*Adgrv1* ^*−/−*^ mouse	Progressive HL, disorganisation of HCs, reduced mechanotransduction activity	Not reported	Not reported	Alagramam et al. (2016); Mathur et al. (2023); Michalski et al. (2007)
ADGRV1	*Adgrv1* ^*del7TM*^ mouse	Profound HL by P20, progressive degeneration of OHCs (apical to basal)	Retinal degeneration	Normal	McGee et al. (2006); Linnert et al. (2023)
ADGRV1	*Adgrv1 Y6236fsX1* mouse	Progressive disorganisation of HC stereocilia, complete loss of HCs by P28, reduced mechanotransduction activity	Not reported	Normal	Guan et al. (2023)
ADGRV1	*adgrv1* ^*rmc22*^ zebrafish	Normal	Retinal degeneration	Not reported	Stemerdink et al. (2023)
WHRN	*Whrn* ^*wi*^ *(whirler)* mouse	Profound HL, shortened stereocilia in IHCs and rounded stereocilia in OHCs	Normal	Circling and head-bobbing behaviours	Holme et al. (2002); Mburu et al. (2003)
WHRN	*Whrn* ^*tm1Tili*^ *(or Whrn* ^*neo*^ *)* mouse	Moderate HL, disorganised and shortened stereocilia	Absence of long whirlin isoform resulted in reduced usherin expression in all photoreceptors	Normal	Yang et al. (2010)
CLRN1	*Clrn1* ^*−/−*^ mouse	Early-onset profound HL, fragmented stereocilia, elevated ABR thresholds	Early onset of vision loss	Vestibular dysfunction	Geng et al. (2009); Geng et al. (2017)
CLRN1	*Clrn1 p.N48K* mouse	Progressive HL	Progressive vision loss	Variable vestibular dysfunction	Geng et al. (2012)
CLRN1	*Clrn1* ^*−/−*^ zebrafish	Altered HC organisation with splayed stereocilia	Progressive vision loss, disrupted outer segment, degeneration of rod and cone photoreceptors, reduced electrophysiological response	Abnormal swimming behaviour or orientation, diminished hair bundle integrity with splayed stereocilia	Gopal et al. (2015); Nonarath et al. (2024)
CLRN1	*CLRN1* c.143T>C rabbit	Not reported	Early onset of damaged RPE, progressive degeneration of rod and cone photoreceptors	Not reported	Yang et al. (2024a)
CLRN1	*Clrn1* ^*−/−*^ pig	Not reported	Declining rod photoreceptor function from 6 months	Not reported	Dinculescu et al. (2022)

Mouse models have been useful for modelling the phenotype of various USH gene mutations *in vivo*, as mutations induced in different regions of the same gene can yield varying effects on mouse phenotype as in humans. Miller et al. (2012) reported different phenotypes in *Ewaso* (p.I487N) and *Dumbo* (p.F947I) mice, where the former model had profound HL with circling behaviour indicative of vestibular dysfunction and the latter mice had progressive HL without a vestibular phenotype. The *Ewaso* model would thus be a more appropriate model for USH1B, while the *Dumbo* model appears to match the phenotype for DFNB2, a non-syndromic hearing disorder. Moreover, the c.2839T>A mutation in the *Dumbo* model affects the linker region of MYO7A, while the c.1460T>A *Ewaso* mutation affects the head domain. Mutations in the head domain have shown a deafness and vestibular dysfunction phenotype like the *shaker-1* (c.1505G>C) and *Headbanger* (c.531A>T) models, and likely have the greatest effect on phenotype due to the head domain being essential for myosin protein function (Gibson et al., 1995; Rhodes et al., 2004).

Despite their utility for studying the hearing and vestibular phenotype of USH, the majority of mouse models do not show an abnormal vision phenotype (Williams, 2008). The *shaker-1* mouse for example, exhibits progressive HL, degeneration of the organ of Corti and behaviours indicative of vestibular dysfunction. However, their vision is only mildly impaired, though this was found to be exacerbated by light exposure (Peng et al., 2011). Some theories have suggested functional redundancy, alternative splicing, the higher ratio of rod to cone photoreceptors in mice and slightly differing localisation of USH proteins in the human and mouse retina (Sahly et al., 2012). A more faithful phenotype of disease progression was observed in *Ush2a* ^*−/−*^ mice, which exhibit moderate-to-severe SNHL, stereocilia fragmentation and late-onset, mild retinal degeneration (Liu et al., 2007). There are other rare cases where the mouse phenotype may not exactly align with the human condition. For example, *Whrn* ^*wi*^ (*whirler*) mice also visibly display vestibular dysfunction through circling behaviour, yet USH2 patients rarely have issues with balance (Holme et al., 2002; Mburu et al., 2003; Mustapha et al., 2007).

Significant progress has been achieved in the characterisation of new zebrafish models. Zebrafish are particularly useful due to their transparent bodies, large eyes and additional presence of HCs along a longitudinal strip of their bodies known as the lateral line. Additionally, more zebrafish models of USH have retinal degeneration phenotypes than their mouse counterparts. USH models created in other animals including rabbits and pigs have also been reported recently (Table 1). These were created in the absence of a retinal phenotype for a given subtype in mice and zebrafish and are advantageous in their closer anatomical and physiological resemblance to humans in the case of the pig (Nguyen et al., 2023a). A non-human primate model of USH1B generated by CRISPR-Cas9 was recently reported, however editing efficiency was insufficient to create a disease phenotype in the animals (Ryu et al., 2022).

Despite the obvious anatomical, physiological and genetic differences between human and animal model in disease progression, animal models remain important for elucidating the disease mechanism of USH subtypes and generating novel treatments. The more translationally relevant animal models will have a similar mutation to the orthologous human gene and expected phenotype. Moreover, the spatiotemporal expression of some USH genes have been comprehensively mapped in mice, with one study by Kolla et al. (2020) showing the expression of genes such as *Myo7a*, *Pcdh15* and *Cdh23* at various timepoints between E14 and P7 in mice. This will allow for the better design of gene therapies that can be transferred or redesigned for human use. Moreover, animals remain essential for standard preclinical studies of drug delivery, dosage and safety for any new therapy of USH.

## 10 Precision medicine for Usher syndrome

Precision medicine involves the use of patient data to refine therapeutic options and decisions to suit an individual patient’s condition. Comprehensive data such as; genetics, omics, lifestyle, demographics, physiological measurements and comorbid conditions of the patient, combined with clinical and therapeutic outcomes are shared and databased. This information can be used to refine diagnostic and therapeutic choices of future patients to allow faster intervention and improve clinical and therapeutic outcomes (König et al., 2017; Kosorok and Laber, 2019).

The application of precision medicine could improve diagnosis and treatment outcomes for SNHL (Ginsburg and Phillips, 2018; Wafi and Mirnezami, 2018). Genetic screening could be coupled with existing newborn hearing screening programs to improve diagnostic efficiency, improve detection of genetic SNHL and monitor progression of hearing impairment in USH individuals (Wang et al., 2011b; Wu et al., 2017; Shearer et al., 2019). A necessity of precision medicine is the compilation and computation of a vast amount of patient and population data for which suitable infrastructure is required (Ginsburg and Phillips, 2018; Wafi and Mirnezami, 2018). The Usher Syndrome Coalition is an organisation working to support individuals and families living with USH (Usher Syndrome Coalition, 2025). This along with biobanks such as the Australasian Hearing Registry and Biobank, and the Western Australian Retinal Degeneration study biobank aim to improve understanding of genotype-phenotype correlation; improve communication between the scientific community, patients and their families and; facilitate researcher access to patient samples and clinical data. Advancing understanding of gene therapy strategies, population genetics and the structure and functions of the USH genes will aid in developing methods for restoring function in USH proteins to treat those affected by it (Redfield et al., 2025).

### 10.1 Preclinical Usher patient-specific induced pluripotent stem cell and organoid models

Since human induced pluripotent stem cells (iPSCs) were first generated by the Yamanaka group, iPSCs have become an invaluable tool for modelling many human diseases and as a potential source for cell therapy (Takahashi and Yamanaka, 2006). iPSCs can be generated from non-invasively collected somatic cell types including dermal fibroblasts, peripheral blood mononuclear cells (PBMCs), keratinocytes and urine-derived cells (Aasen et al., 2008; Loh et al., 2009; Zhou et al., 2011; McLenachan et al., 2019; Zaw et al., 2021; Wong et al., 2024).

Recently, protocols for the differentiation of inner ear and retinal organoids from iPSCs have emerged (Eiraku et al., 2011; Zhong et al., 2014; Koehler et al., 2017; Jeong et al., 2018). Over time protocols have been adapted to specifically manipulate signalling pathways, such as Fgf, BMP, TGFβ, Wnt and Sonic Hedgehog at various timepoints during organoid growth (Zhou et al., 2015; Lahlou et al., 2018). This is done to mimic the early growth of the human inner ear and provides the opportunity to study the role of genes and diseases during development. These protocols could finally allow investigation of USH disease pathophysiology in a human model. These organoid models have been characterised to grow cochlear, vestibular and retinal cell types (Finkbeiner et al., 2022; Doda et al., 2023; Moore et al., 2023; Steinhart et al., 2023; Tresenrider et al., 2023). Moreover, protein interactions key to USH mechanisms could be modelled. The reported iPSC lines generated from USH1B and USH2A patients with mutations in *MYO7A* and *USH2A* have been summarised in Table 2 iPSC lines for other USH subtypes are yet to be reported.

**TABLE 2:** Reported iPSC lines generated from patient tissues.

Causative gene	Genotype	Mutations	Exon region	Domain affected	Cell source	Reference
MYO7A	Homozygous	c.496del (p.E166Rfs*5)	Exon 6	Motor domain	Dermal fibroblasts	Wong et al. (2024)
USH2A	Heterozygous	Pseudoexon 40 (c.7595–2144A>G) c.12575G>A (p.R4192H)	Intron 40 Exon 63	Fibronectin type III 27	Keratinocytes	Tucker et al. (2013)
USH2A-RP	Homozygous	c.8559–2A>G (p.Y2854_2894Rdel)	Intron 42	Fibronectin type III 15	Urine-derived cells	Guo et al. (2018)
USH2A	Homozygous	c.2299delG (p.E767Sfs*21)	Exon 13	Laminin EGF-like 5	Dermal fibroblasts	Sanjurjo-Soriano et al. (2018a)
USH2A	Compound heterozygous	c.2276G>T (p.C759F) c.2299delG (p.E767Sfs*21)	Exon 13	Laminin EGF-like 5	Dermal fibroblasts	Sanjurjo-Soriano et al. (2018b)
USH2A	Homozygous	c.2276G>T (p.C759F)	Exon 13	Laminin EGF-like 5	Dermal fibroblasts	Zurita-Díaz et al. (2018)
USH2A-RP	Compound heterozygous	c.2209C>T (p.R737X) c.8693A>C (p.Y2898S)	Exon 13 Exon 44	Laminin EGF-like 4 Fibronectin type III 15	Dermal fibroblasts	Riera et al. (2019)
USH2A	Compound heterozygous	c.949C>A (p.Y318Cfs*17) c.1256G>T (p.C419F)	Exon 6 Exon 7	Laminin N-terminal	Dermal fibroblasts	McLenachan et al. (2019); Zaw et al. (2021)
USH2A	Homozygous	c.2299delG (p.E767Sfs*21)	Exon 13	Laminin EGF-like 5	Dermal fibroblasts	Dulla et al. (2021)
USH2A	Compound heterozygous	c.1256G>T (p.C419F) c.2299delG (p.E767Cfs*21)	Exon 7 Exon 13	Laminin N-terminal Laminin EGF-like 5	PBMCs	Liu et al. (2021)
USH2A-RP	Compound heterozygous	c.2802T>G (p.C934W) c.12560G>A (p.R4187H)	Exon 13 Exon 63	Laminin EGF-like 8	PBMCs	Zhu et al. (2021)
USH2A	Homozygous	c.8559-A>G (p.Y2854_2894Rdel)	Intron 42	Fibronectin type III 15	PBMCs	Zhu et al. (2021)
USH2A	Homozygous	c.8559–2A>G (p.Y2854_2894Rdel)	Intron 42	Fibronectin type III 15	PBMCs	Liang et al. (2022)
USH2A-RP	Compound heterozygous	c.2276G>T (p.C759F) c.7352T>C (p.L2451P)	Exon 13 Exon 39	Laminin EGF-like 5	Dermal fibroblasts	Sanjurjo-Soriano et al. (2022)
USH2A	Homozygous	c.8559–2A>G (p.Y2854_2894Rdel)	Intron 42	Fibronectin type III 15	PBMCs	Chen et al. (2023)
USH2A	Compound heterozygous	c.538T>C (p.S180P) c.2802T>G (p.C934W)	Exon 3 Exon 13	Laminin G-like 2 Laminin EGF-like 8	PBMCs	Qiu et al. (2023)
USH2A	Compound heterozygous	c.1907_1912ATGTTT>TCACAG (p.D636V + V637T + C638G) c.8328_8329delAA (p.L2276fs*12)	Exon 11 Exon 42	Laminin EGF-like 2 Fibronectin type III 14	PBMCs	Ukaji et al. (2023)

#### 10.1.1 Usher syndrome type 1B

Recently, Gilmore et al. (2023) studied the expression of MYO7A isoforms in retinal organoids, which were generated from a donor with no known retinal disease and a commercially available episomal human iPSC line (Buchholz et al., 2013; Zhong et al., 2014; Hazim et al., 2017; Aparicio-Domingo et al., 2023). The organoids exhibited similar RPE features as human RPE, including critical protein expression, tight junction formation and phagocytosis of photoreceptor outer segments. Three-dimensional modelling of the two MYO7A isoforms showed differences in amino acid sequences at the tip of a loop of the FERM1 domain, which may result in changes in protein interaction. Interestingly, while both isoforms were expressed in human retinal organoids, mouse and pig RPE and neural retina, the proportion of the isoforms varied between these studied species. For instance, the human retinal organoid and pig models express similar levels of MYO7A short isoform at 82%–90%, while the long isoform is predominantly found in the mouse retina (Gilmore et al., 2023). This study demonstrates how iPSC can be used in situations where; animal models are known to differ from human genetics or physiology; to better understand the precise differences between human and animal disease models; and when human tissue types are inaccessible or scarce.

Leong et al. (2022) observed changes in retinal phenotype in iPSC-derived USH1B retinal organoids from three patients with *MYO7A* mutations. The organoids were found to be at a developmental stage equivalent to human foetal retina. The organoids did not show any cellular degradation but displayed heightened expression of genes related to adaptive stress response and apoptosis. Rod photoreceptors displayed upregulated expression of a pro-apoptotic factor (BNIP3), antioxidant enzymes (PRDX1, PRDX2, and PRDX5), and a free radical scavenging enzyme (SOD1). Apoptosis and stress response-related processes were also differentially expressed in Muller cell and bipolar cells. This stress response may be a primary factor in retinal degeneration caused by mutations in *MYO7A,* but upregulation of apoptotic pathways may indicate that Muller and bipolar cells should be further investigated for their role in USH1B pathophysiology (Leong et al., 2022).

#### 10.1.2 Usher syndrome type 2A

In a study by Tucker et al. (2013), keratinocytes from a 62-years old USH2A-RP patient carrying missense mutation c.12575G>A and deep-intronic mutation intron 40 (pseudoexon 40) were reprogrammed into iPSCs and then differentiated into rod photoreceptor precursor cells (PPCs) (Tucker et al., 2013). These retinal-like cells developed eyecup-like structures with a layer of RPE and non-pigmented neural retina, sharing structural features with human retinal precursor cells. Surprisingly, Tucker et al. (2013) did not report any notable morphological changes between USH2A and normal retinal-like cells.

Recently, Guo et al. (2019) generated three-dimensional retinal organoids from USH2A-RP patient iPSC with compound heterozygous mutations c.8559–2A>G and c.9127-9129delTCC in *USH2A*. The authors described USH2A patient retinal organoids as being morphologically smaller and had abnormal retinal formation compared to control organoids. Layers of neural retina and RPE were observed in organoids by day 34 of culture with normal RPE-like cells displaying a cobblestone-like morphology with pigmentation. Conversely, USH2A patient retinal organoids lacked pigmentation due to absence of melanin and showed signs of cellular degeneration due to RPE atrophy. Indeed, expression of apoptotic genes were also significantly higher than controls on day 34 (Guo et al., 2019). Moreover, gene expression of retinal developmental markers were significantly reduced at day 18 in USH2A retinal organoids compared to control. Moreover, the patient organoids had decreased expression of ankle link complex-related genes, including *PDZD7* (Guo et al., 2019).

Similarly, two USH2A patient cell lines with either one or two copies of the c.8559–2A>G mutation in *USH2A* were used to generate retinal organoids in a microfluidic system (Su et al., 2022). By day 18 of differentiation, expression level of pro-apoptotic protein BAX was significantly elevated in USH2A retinal organoids while anti-apoptotic protein BCL2 expression had decreased when compared to normal control organoids. These patient retinal organoids also have reduced laminin and collagen type IV expression affecting integrin expression and thereby downregulating PI3K-Akt signalling, an essential pathway for regulating cell proliferation and growth. Furthermore, expression of cytoskeleton organisation proteins were disrupted in USH2A retinal organoids, suggesting mutations in *USH2A* are linked to impaired extracellular functions causing cell apoptosis in the retina (Su et al., 2022).

## 11 Developing therapies

Developing therapies to date have largely targeted either hearing or vision loss rather than both senses, with recent clinical trials mostly focused on treatment of RP. This could be due to HL preceding the onset of RP, giving a wider treatment window for vision loss than hearing. Another consideration is drug delivery, as the retina is easier to access than the inner ear. Drug delivery to the inner ear would require cochleostomy for direct application to the cochlea or transtympanic injection to apply the drug to the round window niche. The latter method however, assumes passive diffusion of the drug across the round window membrane and this is governed by numerous chemical properties of the drug (Hao and Li, 2019; Delaney et al., 2023).

**FIGURE 3:**
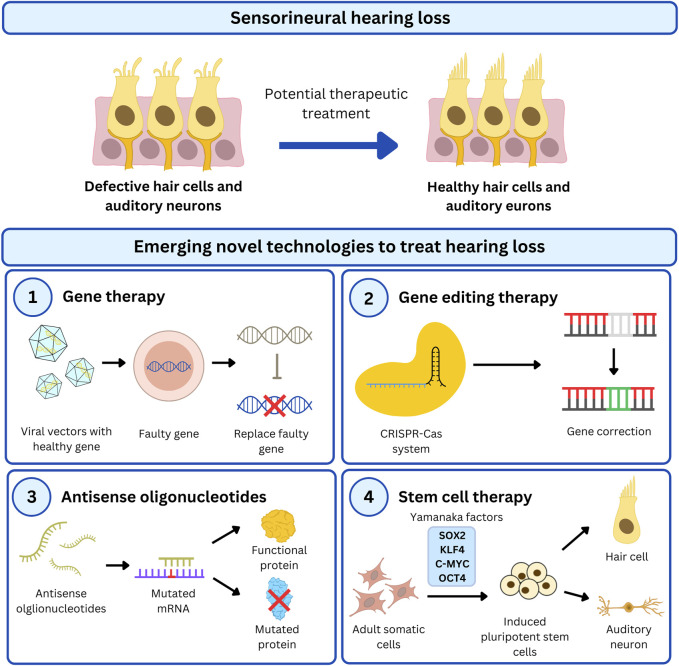
Overview of emerging therapeutic strategies to treat HL in USH patients. USH genes are a major cause of untreatable genetic HL and has recently been targeted by cutting-edge technologies to restore functional HCs in the cochlea. There are different approaches used in preclinical studies to restore hearing which includes gene- and cell-based therapy. Gene therapy is a popular treatment option to correct faulty genes by (1) introducing healthy gene transcripts via viral vectors, (2) precisely correct the genome using the CRISPR-Cas system, or (3) modify protein function by modulating gene expression with antisense oligonucleotides. Additionally, stem cells can be genetically modified with gene-targeted therapy for (4) cell therapy to replace compromised cells.

In this section, we will discuss gene augmentation, gene editing, drug therapy and cell therapy development for various USH subtypes (Figure 3) which are summarised in Tables 3–5.

**TABLE 3:** Gene augmentation therapy for USH genes in preclinical models.

Gene	Model	Target site	Type of viral vector	Outcome	References
*MYO7A*	*Myo7a* null mice	Retina	Single and dual AAV vector	Single AAV vector has better delivery efficiency than dual AAV vector Both restored *Myo7a* expression in the retina, corrected melanosome and ciliary opsin localisation	Lopes et al. (2013)
*MYO7A*	*shaker-1* mice and pig	Retina	Dual AAV and fragmented AAV vectors	Restored Myo7a expression in photoreceptors, corrected melanosome localisation	Trapani et al. (2014)
*MYO7A*	Mice	Inner ear	Fragmented AAV (fAAV) vector	Limited cellular uptake and transduction of dual AAV carrying full-length *MYO7A* cDNA	Dyka et al. (2014)
*MYO7A*	Mice	Retina	Lentiviral vector	Restored *Myo7a* expression in the retina, protected photoreceptors from intensity light damage and improved transduction	Zallocchi et al. (2014)
*MYO7A*	Mice	Inner ear and retina	Dual AAV8 vector	Restored *Myo7a* expression in the inner ear, improved HC survival but cochlear stereocilia remained highly disorganised	Lau et al. (2023)
*MYO7A*	Mice and primates	Retina	Dual AAV8 vector	Specifically biodistributed into ocular tissues, assessed safety and pharmacokinetics of gene therapy for clinical translation use	Ferla et al. (2023)
*MYO7A*	*shaker-1* mice	Inner ear	Lentiviral vector	Improved hearing and balancing function in P90 treated mice although damage in OHC was still observed at the basal turn at 6 months of age	Schott et al. (2023)
*USH1C*	Mice	Inner ear	Anc80L85 vector	Recovered *Ush1c* expression levels, restored HC function and morphology close to WT mice	Pan et al. (2017)
*CDH23* (DFNB12)	Mice	Inner ear (cochlea)	Triple AAV vectors	Limited transduction in IHCs with limited improvement in HC morphology and hearing function	Yoshimura et al. (2023)
*PCDH15* (p.R245X)	Mice	Inner ear	Mini-PCDH15 AAV vector	Improved ABR thresholds and partially rescued cochlear HCs	Ivanchenko et al. (2023)
*SANS*	Mice	Inner ear	AAV vector	Restored full-length *Sans* expression in cochlear HCs, restored hair bundle morphology and number of stereocilia	Emptoz et al. (2017)
*WHRN*	Mice	Retina	AAV vector	Restored whirlin expression and localisation at 2 weeks for up to 6 months post-treatment in the retina	Zou et al. (2011)
*WHRN* (DFNB31)	*Whirler* mice	Inner ear	AAV vector	Restored whirlin expression in the inner ear, restored stereocilia length and improved hearing at all frequencies	Chien et al. (2016)
*WHRN*	Neonatal *whirler* mice	Inner ear	AAV vector	Restored whirlin expression in the inner ear, and restored balancing function and improved hearing for at least 4 months	Isgrig et al. (2017)
*CLRN1*	Mice knockout	Retina	AAV vector	Restored *Clrn1* expression in the retinal cells and neurons in adult mice	Dinculescu et al. (2016)
*CLRN1*	Mice	Inner ear	AAV2/8 vector	Restored *Clrn1* expression in the inner ear, displayed normal hearing through adult life and preserved hair bundle structure	Geng et al. (2017)

**TABLE 4:** Gene editing therapy for USH in preclinical models.

Gene (mutation)	Model	Target site	Intervention	Outcome	References
*MYO7A* (c.4118C>T)	iPSC-derived HC-like cells	Inner ear	CRISPR-SpCas9	Restored *MYO7A* protein expression, improved stereocilia organisation in HC-like cells with electrophysiological properties	Tang et al. (2016)
*USH1C* (p.R31X)	Mice	Inner ear	ZFN	Restored harmonin expression level	Overlack et al. (2012)
*USH2A* (c.2299delG)	Fibroblast	Retina	CRISPR-Cas9	Corrected point mutation in patient fibroblasts	Fuster-García et al. (2017)
*USH2A* (c.2276G>T; c.2299delG)	iPSCs	Retina	CRISPR-eSpCas9	Corrected point mutation in patient iPSCs	Sanjurjo-Soriano et al. (2020)
*USH2A* (c.1256G>T; c.2299delG)	iPSCs	Retina	CRISPR-Cas9	Corrected c.2299delG mutation by adding a guanine nucleotide back into genome in patient iPSCs	Liu et al. (2021)
*USH2A* (c.2209C>T)	iPSCs	Retina	CRISPR-Cas9 and ZFN	ZFN-mediated correction c.2209C>T was more efficient than CRISPR-mediated gene editing	Siles and Pomares (2025)
*CLRN1* (c.256–648T>G)	*in vitro* retinal cell models	Retina	CRISPR	Removed mutation by replacing the aberrant splice donor site with complementary DNA sequence in human retinal cell lines	Panagiotopoulos et al. (2020)

**TABLE 5:** Antisense oligonucleotide-based therapy for USH in preclinical models.

Gene (mutation)	Model	Target site	Outcome	References
*USH1C* (c.216G>A)	Mice	Inner ear	Restored *Ush1c* mRNA transcript levels comparable to WT mice, improved hearing function in mice (local RWM injection at P1)	Lentz et al. (2020)
*USH2A* (c.7595–2144A>G)	Fibroblast	Retina	Restored *Ush2a* mRNA transcript levels in patient fibroblasts	Slijkerman et al. (2016)
*USH2A* (c.4338_4339delCT)	Zebrafish and human organoid	Retina	Restored functional usherin by inducing exon skipping in exon 19-20	Phillips et al. (2024)
*USH2A* (c.2299delG)	Zebrafish	Retina	Restored usherin expression by inducing skipping of murine exon 12 to produce shortened usherin, preserved HC morphology and hearing function	Dulla et al. (2021)
*USH2A*	Zebrafish	Retina	Proof-of-concept by restoring reading frame to produce functional usherin by skipping exon 30-31 or exon 39-40	Schellens et al. (2023)
*USH2A* (c.1551–504C>T; c.1841–377A>G; c.4397–3890A>G; c.4885 + 375A>G)	Minigene assays and patient iPSC-derived PPCs	Retina	Redirected aberrant splicing caused by mutations in introns 8, 10, 20 and 23 in minigene assays Redirected aberrant splicing in introns 8 and 20 in patient-specific PPCs	Reurink et al. (2023)
*USH2A*(c.8681 + 3960A>G; c.9958 + 3438A>G; c.14134–3169A>G)	Minigene assays	Not specified	Redirected aberrant splicing, prevented PE inclusion in the coding sequence at doses 5–50 nM	García-Bohórquez et al. (2024)
*CLRN1* (c.256–649T>G)	Wildtype mice and *in vitro* retinal cell models	Retina	Restored native *Clrn1* mRNA transcript by blocking mutation in intronic 0b region in humanised mice and human retinal cell lines	Panagiotopoulos et al. (2020)

### 11.1 Gene replacement therapies

Gene replacement therapy, or gene augmentation, is the delivery of a functional copy of the affected gene, which can be delivered by viral or non-viral methods. Recent progress in gene augmentation has shown promising outcomes in preclinical studies which has been summarised in Table 3, with some advancing into clinical trials (Table 6). Adeno-associated viruses (AAV) are widely used as a delivery vector as they are not associated with any human disease and are essentially inert without cargo. The use of viral vectors can be limited by the packaging capacity of the vector. AAV vectors are generally constrained to 5 kb such that genes that exceed this capacity, such as several USH genes, require alternative methods to allow their delivery. Despite the capacity limit AAVs have been used to deliver USH genes, for example, rAAV2/8 has been used to deliver *Sans* and Anc80L65 for *Ush1c* early postnatal knockout mice which improved hearing and restored vestibular function (Emptoz et al., 2017; Pan et al., 2017).

**TABLE 6:** Current clinical trial status of gene and cell therapy interventions for USH-associated disorders.

Disease (gene)	Study phase and status (clinical trial ID)	Intervention (drug name)	Target site	Route of administration and treatment method
USH1B (*MYO7A*)	Phase 1/2, terminated (NCT01505062)	Lentiviral vector (SAR421869)	Retina	Single subretinal injection at 1.4^10x5^, 4.7^10x5^, 1.4^10x6^ transducing units in cohort 1, 2 and 3
USH1B (*MYO7A*)	Phase 1/2, recruiting (NCT06591793)	Dual AAV (AAVB-081)	Retina	Single subretinal injection at various dosage
USH2A (*USH2A*)	Phase 1/2, completed (NCT03780257)	ASO (QR-421a)	Retina	Single intravitreal injection at doses 50, 100, 200 µg
USH2A (*USH2A*)	Phase 2, recruiting (NCT06627179)	ASO (QR-421a)	Retina	Single intravitreal injection at 180 µg (3.6 mg/mL) on day 1 and 60 µg (1.8 mg/mL) on 6, 12, 18 months
USH3A (*CLRN1*)	Phase 1, not yet recruiting (NCT06592131)	Small molecule (BF844)	Inner ear and retina	Oral administration, single versus multiple-ascending doses
All USH genes	Phase 1/2, active (NCT04355689)	Small molecule (NPI-001)	Retina	Oral administration, 250 mg tablet twice a day
RP-associated genetic disorder	Phase 1/2, completed (NCT02320812)	Human iPSC-derived retinal progenitor cell (jCell)	Retina	Single intravitreal injection at various dosage (0.5–3 million cells)
RP-associated genetic disorder	Phase 2, completed (NCT03073733)	Human iPSC-derived retinal progenitor cell (jCell)	Retina	Single intravitreal injection at 3–6 million cells
RP-associated genetic disorder	Phase 2, completed (NCT04604899)	Human iPSC-derived retinal progenitor cell (jCell)	Retina	Single intravitreal injection of 6 million retinal progenitor cells
RP-associated genetic disorder	Phase 1, recruiting (NCT04284293)	Human iPSC-derived astrocytes (CNS10-NPC)	Retina	Single, unilateral, subretinal injection at various dosage
RP-associated genetic disorder	Phase 1/2, not yet recruiting (NCT06789445)	Human iPSC-derived PPCs (OpCT-001)	Retina	Single subretinal injection at various dosage

AAVs are thought to provide transgene expression even in non-replicating cells as they persist as extrachromosomal DNA. Moreover, this lack of integration has the advantage as it minimises safety concerns of insertion into the genome causing deleterious effects due to disruption of the genes at the insertion site. However, the stability of expression is likely to be impacted by any immune response mounted against the viral vector or delivered transgene (Li and Samulski, 2020). Inhibition of transduction and expression of the delivery product and damage due to inflammatory responses are all factors affected by the immune response to gene therapy treatments that must be considered. However, there is continued research into ways to mitigate the immune response elicited by viral vector delivery such as modification of viral vector such as the virus capsids to evade immune response and immunomodulation (Shirley et al., 2020).

The use of specific serotypes and promoters have been investigated to achieve gene delivery specific to targeted cell types, for example, AAV serotype 2/8 has been used to target IHCs and AAV5 is known to transfect photoreceptors and RPE (Zou et al., 2011; Chien et al., 2016; Isgrig et al., 2017). Zou et al. (2011) further used the human rhodopsin kinase promoter to specify expression to photoreceptor cells and attempt to mimic the low abundance expression level of wildtype whirlin. The use of serotypes known to selectively transduce specific cell types and promoters that mimic endogenous expression of the delivered gene can reduce off-target effect of gene therapy and concerns of overexpression of the delivered genes improving safety of future prospective treatments.

One strategy to address genes that are too large for delivery in a standard AAV method includes fragmented genome (fAAV) delivery. An oversized gene is packaged into a single AAV vector resulting in fragmentation of the gene which is then reassembled via recombination of the full-length cDNA upon delivery to the cell. While delivery of full-sized MYO7A has been achieved by fAAV overall this approach has been limited by low vector titers and transduction efficiency (Lopes et al., 2013; Akil, 2020). Additionally, the randomised manner of gene fragmentation on insertion could lead to a heterozygous payload across the vector preparation.

Multi-vectors would avoid this complication as the predetermined sectioning of the gene split across multiple AAV vectors would ensure a consistent payload across the vector preparation. Once the AAV vectors are transduced, the gene segments are joined by one of several strategies; overlapping, trans-splicing or a hybrid of the two approaches. The use of multiple vectors may hamper transduction with dual and triple vector approaches reporting reduced efficiency compared to single AAV delivery (Colella et al., 2014; Carvalho et al., 2017; Maddalena et al., 2018; Yoshimura et al., 2023).

A promising dual vector approach was recently tested for USH1B, with preclinical animal studies in both the inner ear and retina. Dual vectors are able to mediate expression of full-length MYO7A with efficiency equivalent or surpassing fAAV (Dyka et al., 2014). Lau et al. (2023) tested dual vector AAV8 delivery of *MYO7A* to the inner ear of *shaker-1* mice. Although cochlear HC morphology and auditory function were not rescued, improved vestibular HC morphology, vestibular sensory-evoked potential threshold and reduced circling behavior were observed. This dual vector approach may be viable for the treatment of vestibular function in USH1B. Ferla et al. (2023) assessed pharmacokinetics and safety of good-manufacturing-practice-like AAV8.MYO7A dual vector of low and high doses delivered subretinally to *shaker-1* mice and non-human primates. Administration resulted in expression of MYO7A protein and improved melanosome localisation in *shaker-1* mice. In non-human primates, biodistribution of the vector was localised to the retinal and ocular tissues with minimal detection, aside from in serum and the lymphatic system with no detectable spread to the gonads. Overall data reported no major adverse effects (Ferla et al., 2023). These preclinical studies using the dual vector approach have culminated in a Phase I/II clinical trial (NCT06591793) for USH1B-related RP. This clinical trial is sponsored by AAVangard Bio and this study aims to evaluate the safety and efficiency of a single subretinal administration in 15 patients over 61 months.

Another strategy to compensate for large gene delivery is mini-genes, in which versions of the gene are designed without non-essential regions to enable delivery in a single AAV vector. For example, a mini-PCDH15 gene delivery to *Pcdh15* ^*R245X*^ mice, which carry the orthologous nonsense mutation in USH1F patients, showed improved ABR thresholds and partial rescue of HCs after treatment (Ivanchenko et al., 2023). However, the mini-gene approach is only suitable to genes for which the protein has non-essential sections that can be removed without compromising function.

Other viral vectors in use for gene replacement therapy include lentiviruses, which have a larger carrying capacity of approximately 10 kb. Following the preclinical study for testing and safety evaluation of EIAV-based lentiviral delivery of *MYO7A* in *shaker-1* mice and rhesus macaques, this gene therapy progressed into clinical trials (Zallocchi et al., 2014). The UshStat clinical trial (NCT01505062) study was to assess the safety and tolerability of UshStat when subretinally injected at ascending doses. This study was terminated by Sanofi due to factors not related to safety. There is an active long-term study (NCT02065011) to assess safety, tolerability and biological activity in the 9 participants. In another lentiviral vector-based gene therapy, Schott et al. (2023) demonstrated partial recovery of hearing and full recovery of vestibular function in homozygous *shaker-1* mice. Moreover, lentiviral vectors carrying the full-length *MYO7A* cDNA were able to fully restore auditory and vestibular function in heterozygous *shaker-1* mice. This suggests this treatment may be more effective for other forms of *MYO7A-*associated HL such as DFNB2. While lentiviral vectors are ideal for its larger packaging capacity, low-eliciting immune response and long-term expression, there is a lingering question as to their safety. Lentiviruses can integrate randomly into the host genome and potentially result in gene disruption or mutagenesis.

### 11.2 Gene editing

Gene editing involves correction of mutations in a site-specific manner that retains the endogenous regulation of the gene of interest and have been applied in USH models (Table 4). Older editing strategies include transcription activator-like effector nucleases (TALENs) and Zinc-finger nucleases (ZFNs). ZFNs were previously used to correct a *Ush1c* nonsense mutation, p.R31X, and recover harmonin expression (Overlack et al., 2012). Clustered regularly interspaced palindromic repeats (CRISPR) approaches are considered simpler, cheaper and more efficient and are currently being more widely used than ZFN and TALENs.

Gene editing has similar limitations to gene augmentation as the inner ear remains difficult to access. Additionally, a common delivery method of CRISPR is by viral vector, resulting in complications due to tissue targeting and specificity, efficiency of delivery and immune response. As such advancements in strategies to improve the safety and efficiency of viral vector delivery will benefit both gene editing and gene augmentation.

#### 11.2.1 MYO7A gene editing

Tang et al. (2016) generated three iPSC cell lines from; a deaf patient with compound heterozygous *MYO7A* c.1184G>A and c.4118C>T mutations, his asymptomatic father and a normal donor. The c.4118C>T mutation was corrected using *Streptococcus pyogenes* Cas9 (SpCas9) in iPSC and was differentiated into HC-like cells. Interestingly, stereocilia of these HC-like cells did not conform to the classic staircase-like pattern of mammalian stereocilia, which was proposed to be due to decreased Wnt signalling. When compared with differentiated control lines, MYO7A mutant stereocilia were disorganized and lacked bonding with neighbouring stereocilia, with significant changes to HC electrophysiology. The CRISPR-corrected HC-like cells expressed similar levels of *ATOH1*, *POU4F3*, *MYO7A*, and *ESPN* compared to normal donor HC-like cells. The corrected iPSC produced a full-sized MYO7A protein when analysed by immunoblotting, indicating genetic correction mitigated truncation of the protein. Morphology of the stereocilia showed organisation and bonding comparable to controls. Electrophysiology showed inward and outward currents consistent with HC (Tang et al., 2016).

#### 11.2.2 USH2A gene editing

The CRISPR/Cas9 system was used to correct c.2299delG *USH2A* mutation in fibroblasts with no off-target effects detected (Fuster-García et al., 2017). This was followed by using CRISPR to correct mutations in patient-derived iPSCs wherein Sanjurjo-Soriano et al. (2020) utilised iPSCs from patients with *USH2A* mutations and corrected these mutations using CRISPR editing. Dermal fibroblasts from a homozygous USH2A patient harbouring c.2299delG (Sanjurjo-Soriano et al., 2018a) and a RP-associated patient with compound heterozygous mutations c.2276G>T and c.2299delG in *USH2A* (Sanjurjo-Soriano et al., 2018b) were used to generate iPSCs. Both USH2A iPSC lines were corrected using the enhanced specificity SpCas9 (eSpCas9) with high targeting efficiency and did not induce off-target effects. These CRISPR-corrected iPSC lines maintained pluripotency as they expressed similar levels of OCT3/4, SOX2 and NANOG compared to their untreated parental iPSC lines.

Liu et al. (2021) collected PBMCs from an USH2A patient harbouring compound heterozygous variants c.1256G>T and c.2299delG to generate iPSCs. Using these iPSCs, they employed CRISPR-Cas9 with a homology repair template to introduce the missing guanine back into the sequence, which they confirmed by DNA sequencing after treatment. The corrected USH2A patient iPSCs had similar pluripotency characteristics and showed the ability to differentiate into the three primary germline layers, suggesting that CRISPR-mediated genome editing did not affect iPSC characteristics.

Recently, gene correction using CRISPR or TALENs technology was compared in iPSCs derived from patients with different genetic forms of inherited retinal disorder (IRD), including USH2A-associated RP. Although CRISPR-mediated gene editing showed superior homology-directed repair correction in other IRD patient iPSCs, TALEN-mediated gene editing efficiency was moderate but higher than CRISPR for correcting *USH2A* c.2209C>T mutation (Siles and Pomares, 2025).

Currently, there are no gene editing-based therapies in clinical trials. However, a CRISPR-meditated therapy for targeting *USH2A* mutations in exon 13, EDIT-102, is being developed by Allergan and Editas Medicine. EDIT-102 comprises the same proprietary enzyme, AAV vector, promoters, and route of delivery as EDIT-101. Currently, EDIT-101 is undergoing the Brilliance clinical trial (NCT03872479) for Leber congenital amaurosis to correct an intronic mutation in *CEP290*, after achieving rapid and sustained editing of somatic non-human primate cells at a level that met the target therapeutic threshold (Maeder et al., 2019).

### 11.3 Drug therapies

USH3A has been proposed to be one of the easier USH subtypes to target for therapeutic intervention. This is owing to its prolonged latency compared to other subtypes and that the causative gene does not encode a structural protein of HCs or photoreceptors. Theoretically therefore, USH3A should have a wider therapeutic window for intervention. Currently, the clinical trial of the antioxidant N-acetylcysteine amide, NPI-001 (NCT04355689) for general USH treatment could be of particular interest for treating USH3A, as animal models have shown improved hearing after antioxidant treatment (Gopal et al., 2019). N-acetylcysteine is currently undergoing a Phase III clinical trial with Johns Hopkins University (NCT05537220) for RP, irrespective of genetic source with 438 participants over 45 months. This follows previous clinical trials in which 6 months of treatment was safe and well-tolerated with improvement of best-corrected visual acuity and macular sensitivity (Campochiaro et al., 2020). Another small molecule drug currently in preclinical development, BF844, has been demonstrated to stabilize the *CLRN1* ^*N48K*^ mutation and attenuate progressive HL in mouse models (Alagramam et al., 2016). The drug is currently undergoing a Phase I clinical trial (NCT06592131) to evaluate pharmacokinetics.

As mentioned previously, upregulation of genes related to apoptosis has been observed in retinal organoids derived from USH patients (Guo et al., 2019; Leong et al., 2022; Su et al., 2022). One explanation for the activation of the apoptosis pathway is the over-accumulation of USH proteins in the endoplasmic reticulum. For instance, MYO7A, harmonin and cadherin 23 are known to localise and assemble a protein complex in the endoplasmic reticulum before being trafficked to the stereocilia (Blanco-Sánchez et al., 2014). A small molecule, Salubrinal, acts to prevent dephosphorylation of eukaryotic translation initiation factor 2 alpha (eIF2α) which has a cytoprotective effect during endoplasmic reticulum stress when phosphorylated (Matsuoka and Komoike, 2015). Salubrinal-treated *erlong* mice, harbouring missense *Cdh23* mutations, had improved ABR thresholds and DPOAE amplitudes and OHC survival after treatment (Hu et al., 2016). These encouraging results indicate endoplasmic reticulum stress may be an interesting novel target to delay HC death for some USH types, and extend the treatment window for other therapeutic strategies that require intact HCs.

#### 11.3.1 Antisense oligonucleotide therapies

Antisense oligonucleotide (ASO) therapy is an alternative method to treat inherited disorders like USH by using a synthetic 21–25 single-stranded oligodeoxynucleotide that complementarily bind to the target mRNA to regulate gene expression (Rinaldi and Wood, 2018). ASO-based drugs have proven to be a promising therapeutic approach to target specific genetic diseases such as Duchenne muscular dystrophy, which restored functional mRNA transcripts of the *DMD* gene (Lauffer et al., 2024). Fortunately, development of ASO treatments is rapidly advancing in preclinical studies for USH genes with promising outcome as summarised in Table 5.

##### 11.3.1.1 USH1C antisense therapies

Preclinical applications of drug candidate ASO-29 to target Acadian mutation c.216G>A in exon 3 of *USH1C* for the inner ear have showed promising results. Mice harbouring murine equivalent c.214G>A mutation treated at different dosage and inner ear HC developmental stages have various levels of hearing restoration (Lentz et al., 2013; Lentz et al., 2020; Wang et al., 2020). In Lentz et al. (2020) study, ASO-29 was directly administered in P1 Ush1c^216AA^ mice via local round window membrane injection. Treated mice had significantly improved hearing thresholds and cochlear hair bundle morphology. Currently, the Lentz group are actively conducting natural history studies of USH1C patients to investigate the disease progression and identify suitable participants for future clinical trials (Lentz et al., 2023). Their earlier study also enlightened another possibility for treating visual impairment caused by *USH1C* c.216G>A, where significant improvement in visual function and retinal structure was shown in neonatal mice when ASO was locally administered to the eye (Amato et al., 2015). In 2019, the Lentz and Koenekopp groups collaborated to investigate potential ASO treatment to target vision loss caused by *USH1C* c.216G>A, where in their most recent work, they reported mRNA transcript levels of corrected *Ush1c* significantly increased in treated mutant mice by modifying the chemical moiety of the parent drug ASO-29 (Alapure et al., 2023; Alapure et al., 2024).

##### 11.3.1.2 USH2A antisense therapies

There are an increasing number of newly identified deep-intronic variants in *USH2A,* often creating pseudoexon inclusions, premature stop codons or altering the protein function (García-Bohórquez et al., 2024). A deep-intronic c.7595–2144A>G mutation in *USH2A* was first reported in 2012, which results in an insertion of a 152 bp pseudoexon 40 (PE40) into the mature mRNA transcript (Vaché et al., 2012). Fibroblasts from a heterozygous patient with *USH2A* c.7595–2144A>G mutation were treated with ASO to induce splicing correction (Slijkerman et al., 2016). Two different ASOs targeting the PE40 mutation promoted splicing correction in *USH2A* mRNA transcripts which were confirmed by Sanger sequencing. Slijkerman et al. (2016) also transfected both ASOs into patient fibroblasts which achieved higher splicing correction, suggesting a cocktail of ASOs can improve therapeutic outcome. Recently, ASOs targeting deep-intronic mutations in patient iPSC-derived PPCs have shown to be an effective method to correct abberant splicing mutations c.1551–504C>T (PE8) and c.4397–3890A>G (PE20) in *USH2A* (Reurink et al., 2023). Moreover, splicing redirection by ASOs have also been reported for three novel deep-intronic variants in minigene splicing assays (García-Bohórquez et al., 2024).

Multiple exon skipping was recently demonstrated for founder mutation c.4338_4339delCT in exon 20 of *USH2A*, which accounts for 55.6% of USH2 cases in the Quebec French-Canadian population (Ebermann et al., 2009; Phillips et al., 2024). The designed ASO induced in-frame deletion by skipping exons 19 and 20 which together encode for a single fibronectin domain and had showed successful exon skipping potential in a zebrafish, and in human iPSC-derived retinal and inner ear organoid models (Phillips et al., 2024). This effective strategy not only preserved usherin protein folding properties based on an *in silico* analysis but allowing broader application for treating patients with pathogenic variants found in either exon 19 and/or exon 20 (Schellens et al., 2023; Phillips et al., 2024). In a similar study, Schellens et al. (2023) showed simultaneous exon skipping of exons 30-31, or exons 39-40 in *USH2A*, which both encodes for one of the FN3 domains, did not affect its protein function in the zebrafish photoreceptor cells.

In a proof-of-concept study, deletion of murine equivalent exon 12 in *Ush2a* (*Ush2a*Δex12) resulted in a shortened usherin protein. Despite this, *Ush2a*Δex12 mice demonstrated improved hearing function along with preserved HC morphology (Pendse et al., 2019). Exon skipping of the most prevalent c.2299delG mutation in exon 13 of *USH2A* was demonstrated by Dulla et al. (2021) using the ASO, QR-421a. This method of splicing correction also does not disrupt the reading frame for translating *USH2A*, thus producing a shortened usherin protein with residual function. Indeed, QR-421a corrected the *ush2a* transcript reading frame in *ush2a* ^*rmc1*^ zebrafish; the treatment successfully restored some level of usherin protein expression in mutant photoreceptors (Dulla et al., 2021). Moreover, PPCs derived from USH2A patient iPSCs carrying homozygous c.2299delG mutation were treated with QR-421a at concentrations 1–10 µM for 28 days using gymnotic delivery. Exon 13 skipping in *USH2A* was highest at 62% for 10 µM concentration whilst no correction was observed in untreated or control group, indicating high target sequence specificity of QR-421a.

Due to the recent success of Ultevursen (QR-421a) in restoring functional *ush2a* in the zebrafish model, the Stellar trial, a Phase II clinical trial (NCT03780257) was conducted over 24 months with 20 subjects. The study reported that a single intravitreal injection of QR-421a was well-tolerated with stabilisation of visual acuity and improvement in retinal sensitivity and structure (Audo et al., 2022). Since the positive outcome of Stellar trial, the Phase II LUNA trial (NCT06627179) is currently recruiting USH2A-RP patients with mutations in exon 13 to determine the safety and tolerability of QR-421a over the course of 24 months. USH2A-RP patients will initially receive an intravitreal injection of QR-421a at 160 µg and is further administered at 80 µg on 6, 12 and 18 months. This ongoing administration allows the production of partially functional protein by repeatedly inducing skipping of exon 13 in *USH2A* at the mRNA level (Boros et al., 2022; Komaki et al., 2025). For instance, Nusinersen is the only FDA-approved ASO drug for treating spinal muscular atrophy and studies have shown patients require regular administration every 4–6 months at lowered concentration of Nusinersen to maintain levels of corrected protein expression (De Vivo et al., 2019). To mitigate this issue, Ou et al. (2024) introduced an AAV-based gene therapy approach called RM-101 containing SmOPT snRNA and have showed high efficiency of skipping exon 13 in *USH2A*. Moreover, humanised mice treated with RM-101 have sustained *USH2A*Δex13 transcript expression and did not show any retinal abnormalities, offering a different perspective to treat USH2A-associated RP.

## 12 Cell therapies

Cell therapy is an approach whereby healthy and functional cells can be transplanted into an individual to replace damaged or lost cells. The application of cell therapy has been thoroughly investigated for regenerative medicine or treating neurodegenerative conditions like USH. Stem cells are widely used due to their self-renewal ability and potency to differentiate into any cell type in the body. There are currently two types of stem cells used for inner ear and retinal cell therapy including embryonic stem cells (ESCs) and iPSCs. Currently, various stem cell products are being developed and tested in clinical trials for RP treatment which has been summarised in Table 6.

jCell is the first allogeneic iPSC-derived retinal progenitor cell product developed by jCyte, which has neuroprotective mechanisms by releasing neurotrophic factors (Yang et al., 2024b). Early results from Phase I/II clinical trial (NCT02320812) have shown jCell to be safe and well-tolerated at various dosages. In the latest Phase IIb study (NCT04604899), participants from the previous Phase II trial (NCT03073733) were re-injected with jCell on the same treated eye and had sustained improvement in visual acuity, contrast sensitivity and kinetic visual fields. Following the success of this clinical trial, jCyte reported they anticipate proceeding into Phase III for jCell.

In 2024, an iPSC-derived neural progenitor cell (NPC) product called CNS10-NPC entered Phase I clinical trials (NCT04284293) for RP. CNS10-NPC was shown to significantly delay photoreceptor degeneration through the promotion of antioxidant effects and release of trophic factors through various signalling pathways in RCS rats modelling retinal degeneration (Lu et al., 2023). Moreover, CNS10-NPC were able to mature into astrocytes and did not show tumorigenicity, making this a promising treatment for RP (Lu et al., 2023). Similarly, the US FDA recently approved fast track designation for OpCT-001, an allogeneic iPSC-derived PPC product developed by BlueRock Therapeutics to treat primary photoreceptor diseases including RP. Currently, a Phase I clinical trial (NCT06789445) is undergoing patient recruitment to investigate safety, tolerability and efficacy of OpCT-001 for 52 weeks.

Rincell-1 by Rinri Therapeutics, is expected to enter clinical trials in 2025. Rincell-1 is a human ESC-derived otic neural progenitor (ONP) product used for regenerating auditory neurons. In their preliminary study, transplantation of human ESC-derived ONP cells into the cochlea of a gerbil model of auditory neuropathy was able to re-establish the synaptic connection with native cochlear HCs, improving hearing ability (Chen et al., 2012). Additionally, Rinri Therapeutics have two stem cell products in preclinical development for hearing restoration, including iPSC-derived ONP (Rincell-2) and otic epithelial progenitor (Rincell-3). While ANP1 (ReSonance) is another neural cell-based product developed by Lineage Cell Therapeutics for treating auditory neuronal disorders, this form of cell therapy does not meet the clinical needs of USH patients. For instance, majority of USH proteins are expressed in HCs and stereocilia, and in USH patients with severely impaired cochlear HCs and auditory nerves, replacing neural cells alone would be insufficient to restore hearing (Sekiya and Holley, 2021).

To date, the only stem cell-based therapy in clinical trial for hearing impairment is the use of autologous mesenchymal stem cells to regenerate HCs in children with acquired SNHL (Baumgartner et al., 2018). Although their results demonstrated the patients had hearing improvement, mesenchymal stem cells have limited differentiation potential and are unable to differentiate into the necessary cell types such as HCs that are missing in USH patients (Pittenger et al., 2019). While these stem cells have restricted ability to only differentiate into neural stem cells, USH patients with preserved HCs could benefit from its immunomodulatory and regenerative properties as mesenchymal stem cells can secrete growth factors and cytokines to preserve HCs as shown in rodent cochlea (Kada et al., 2020; Tsai et al., 2022). Transplanting stem cells that can simultaneously differentiate into both HCs and auditory nerves may be a feasible method to reverse HL in USH patients. Several groups have recently shown iPSCs can differentiate into cochlear sensory epithelium, containing both HCs and neural cells in inner ear organoids, which could potentially provide a renewable source of HCs (Koehler et al., 2017; Moore et al., 2023).

## 13 Conclusion and future therapeutics

Mutations in USH-associated genes affect the sensory cells of the inner ear and retina which do not replenish once damaged. Moreover, USH is still considered an incurable disease, despite being the most common genetic disorder of deaf-blindness. Our understanding of the role that USH proteins play in the inner ear and retina has advanced due to the continued progress in optimising animal models. The USH proteins form an important interactome in the sensory cells of the mammalian inner ear and retina, as they support both the development of sensory cells and their maintenance after birth. Genetic screening and identification of USH mutations combined with patient-derived organoid models will improve our ability to link the functions of USH protein variants with their pathological mechanisms and help identify new therapeutic targets.

The recent success of the clinical trial that delivered a functional copy of the *OTOF* gene with an adeno-associated virus provides hope of further progress in treating genetic HL via gene therapy. *OTOF* gene therapy has been administered in 11 children and improved ABR, speech perception and sound localisation with no dose-limiting toxicity or serious adverse events were reported (Lv et al., 2024; Wang et al., 2024). While these patients will require follow-up to assess long-term effects, it is outstanding progress. It is thought adults may also benefit from similar gene therapy (Qi et al., 2025). One reason that the *OTOF* treatment may be efficient at restoring hearing, is the preservation of the architecture of the organ of Corti and HCs. This is of particular note when considering the treatment of USH3 due to the late onset of the subtype. Genetic screening to identify these patients presents the opportunity to treat their USH with gene replacement or editing therapies before symptoms become too severe.

Cell therapy is a promising strategy to replace defective cochlear HCs and retinal photoreceptor with healthy ones for USH patients, however, there are concerns regarding the safety of stem cells. Manipulating stem cell differentiation is difficult and could potentially lead to undesirable cell types and teratomas (Gunewardene et al., 2014; Isoda et al., 2023). Promisingly, results from clinical trials for retinal disorders using stem cell therapy have yet to report adverse events (Radu et al., 2024). Moreover, most stem cell products in clinical trials are harvested from allogeneic sources and are a popular choice as a functional copy of the gene is readily available. One drawback of allogeneic stem cells is the risk of immune rejection, limiting effective tissue transplantation. Alternatively, autologous stem cells derived from patient’s own cells can mitigate tissue rejection issues, but in USH cases, the dependent on gene therapy will still be necessary.

While we have discussed the new therapeutic strategies individually, there is promising work in other fields where technologies are combined, which could be applicable to USH. Current studies have already shown ways to improve cell transplantation such as knocking out the human leukocyte antigen with CRISPR-Cas gene editing technology (Amiri et al., 2021). Furthermore, transplanting genetically corrected patient iPSCs have already been reported in various inherited disorders including Huntington’s disease and Parkinson’s disease (Cho et al., 2019; Moriarty et al., 2022). Genetically modified stem cells have already been tested in deaf-blind animal models and have been demonstrated to evade immune rejection and successfully improve the engraftment rate of transplanted cells, though a method for transplanting cells in their precise locations, particularly in the organ of Corti has yet to be demonstrated (da Silva et al., 2023; Ishida et al., 2024).

The therapeutic strategies of gene augmentation, gene editing, drug therapy and cell therapy discussed in this review are promising approaches for the treatment of USH. The current and future progress in preclinical and clinical trials will continue to refine these strategies and their delivery, efficacy and safety. The global burden of these sensory deficits has over 1.4 billion people suffering from HL and 1.3 billion from vision loss, with this expected to rise to 2.5 billion people projected to have HL by 2050 (GBD, 2017 Disease and Injury Incidence and Prevalence Collaborators, 2018; World Health Organisation, 2025). The novel therapies discussed in this review and the deeper understanding of the inner ear and retina gained from recent organoid and animal models could be applied to address other causes of hearing and vision loss.
